# Risk factors for antenatal depression, postnatal depression and parenting stress

**DOI:** 10.1186/1471-244X-8-24

**Published:** 2008-04-16

**Authors:** Bronwyn Leigh, Jeannette Milgrom

**Affiliations:** 1 Parent-Infant Research Institute, Department of Clinical and Health Psychology, Heidelberg Repatriation Hospital Austin Health, 300 Waterdale Rd, Heidelberg Heights 3081, Victoria, Australia; 2 Department of Psychology, School of Behavioural Science, University of Melbourne, Victoria, Australia

## Abstract

**Background:**

Given that the prevalence of antenatal and postnatal depression is high, with estimates around 13%, and the consequences serious, efforts have been made to identify risk factors to assist in prevention, identification and treatment. Most risk factors associated with postnatal depression have been well researched, whereas predictors of antenatal depression have been less researched. Risk factors associated with early parenting stress have not been widely researched, despite the strong link with depression. The aim of this study was to further elucidate which of some previously identified risk factors are most predictive of three outcome measures: antenatal depression, postnatal depression and parenting stress and to examine the relationship between them.

**Methods:**

Primipara and multiparae women were recruited antenatally from two major hoitals as part of the *beyondblue *National Postnatal Depression Program [1]. In this subsidiary study, 367 women completed an additional large battery of validated questionnaires to identify risk factors in the antenatal period at 26–32 weeks gestation. A subsample of these women (N = 161) also completed questionnaires at 10–12 weeks postnatally. Depression level was measured by the Beck Depression Inventory (BDI).

**Results:**

Regression analyses identified significant risk factors for the three outcome measures. (1). Significant predictors for antenatal depression: low self-esteem, antenatal anxiety, low social support, negative cognitive style, major life events, low income and history of abuse. (2). Significant predictors for postnatal depression: antenatal depression and a history of depression while also controlling for concurrent parenting stress, which was a significant variable. Antenatal depression was identified as a mediator between seven of the risk factors and postnatal depression. (3). Postnatal depression was the only significant predictor for parenting stress and also acted as a mediator for other risk factors.

**Conclusion:**

Risk factor profiles for antenatal depression, postnatal depression and parenting stress differ but are interrelated. Antenatal depression was the strongest predictor of postnatal depression, and in turn postnatal depression was the strongest predictor for parenting stress. These results provide clinical direction suggesting that early identification and treatment of perinatal depression is important.

## Background

Depression related to child bearing can occur during pregnancy (antenatal depression), after birth (postnatal depression) or both. Antenatal and postnatal depression share similar prevalence ratings to those for depression in the general population with estimates ranging from 12–20%, with a commonly reported estimate of 13% [2-4]. The immediate and longer-term consequences of perinatal depression are far-reaching, affecting not only the mother but her infant, and their relationships. Depression in pregnancy may diminish one's capacity for self-care, including inadequate nutrition, drug or alcohol abuse and poor antenatal clinic attendance, all of which may compromise a woman's physical and mental health and may reduce optimal fetal monitoring or restrict the growth and development of the fetus [5-8]. The consequences of postnatal depression on child development in early infancy, later infancy and early childhood have been the focus of a number of studies, with cognitive, emotional and social development potentially affected [9-11]. The impact on child development is quite modest in high socioeconomic samples and greater when the postnatal depression is chronic and severe [10,11]. The interactional relationship between mother and baby may be compromised in the presence of postnatal depression, the effects of which may be of greater influence than the mere exposure of the infant to maternal depressive symptomatology [10].

Given the high prevalence and serious consequences of antenatal and postnatal depression, efforts have been made to identify risk factors to assist in prevention, identification and treatment. A review of the empirical literature revealed a range of risk factors similar to risk factors for depression at other stages of the lifespan. These and additional risk factors that influence the onset of antenatal and postnatal depression differentially are summarised below.

### Risk factors for postnatal depression (PND)

Three major meta-analytic studies have been conducted revealing a number of risk factors strongly associated with PND: a history of depression, antenatal depression, antenatal anxiety, stressful life events, negative cognitive attributional style, low self-esteem, low social support and low income [4,12,13]. Other risk factors for postnatal depression cited in the literature include young age [14], fewer years of education [15], a history of miscarriage and pregnancy termination [16] and a history of childhood sexual abuse [17].

### Risk factors for antenatal depression

A number of predisposing factors for antenatal depression have been described although there are no reported meta-analyses. Risk factors include young age [18], low income [19], lower educational attainment [18], history of depression [18,20], a history of miscarriage and pregnancy termination [14], and a history of childhood sexual abuse [21], concomitant high anxiety in pregnancy [22], low self-esteem [23] and low social support [24,25]. There appears to be a paucity of research examining major life events and negative cognitive attributional style and their role in antenatal depression.

### Parenting stress

The term 'parenting stress' encompasses the difficulties in adjusting to the parenting role. Previous research has used a broad construct of parenting stress and employed various methods of measurement. These have been inconsistent in their conceptual definition and measurement but do have broad overlap. In this study, we refer to parenting stress as it pertains to the Parenting Stress Index (PSI), outlined in the Measures section. The PSI examines the level of stress within the parent-child system and consists of factors reflecting a parental domain of coping and perceptions of the child. Thus, the subscales assess a range of factors including depression, maternal health, difficult child and difficult parent-child interaction.

In our earlier studies we found that women suffering from postnatal depression were less attached to their infant, found their infants more demanding from 3 months postpartum and that up to 42 months they continued to show significant parenting stress according to the PSI [9,26], and that their interactional difficulties may persist until 3 years postpartum [27]. Thus, understanding the precursors of parenting stress is important because of the potential implications for child development and adjustment, as well as parental adjustment [27-29].

It is unclear however, whether the constellation of risk factors for parenting stress are similar to those identified for perinatal depression. Of other existing evidence, it appears that women with higher parenting difficulties are younger in age, within a lower income bracket and have lower educational attainment [30]. Women with a history of childhood sexual abuse have reported parenting difficulties including feeling less confident and less in control as parents [31], have experienced more stressful life events in the previous month [32], have low social support [33], and a negative cognitive style [34]. However some of these studies have not used validated measures for the construct of parenting stress.

Thus, of the studies that have been published, a similar risk factor profile emerges for antenatal and postnatal depression, although there has been less research conducted on antenatal depression. In addition, the similarity, difference and relationship in risk factor profile for antenatal and postnatal depression and for parenting stress postpartum is yet to be determined. Interestingly, a depressive history, antenatal depression, antenatal anxiety and low self-esteem do not appear to be cited in the research literature as predictors of parenting stress, (although self-esteem has been investigated as a correlate of parenting stress: [35]). In this study, we will also investigate these potential risk factors for parenting stress.

The aim of this study is therefore to further elucidate which risk factors are most predictive of the three outcomes under investigation – antenatal depression, postnatal depression and parenting stress – as well as the relationship between them.

## Methods

### Participants

Participants were primipara and multiparae mothers recruited from antenatal clinics in two major public hospitals in suburban Melbourne, Australia. The antenatal phase comprised 367 participants consecutively recruited over 12 months. For a further two months, the recruitment was biased to include all women screened as depressed (according to EPDS scores above 12.5), but not all women screened with EPDS scores below 12.5. This was a deliberate strategy to increase numbers to further ensure a strong representation of women in the antenatally depressed group in order to facilitate group comparison and to maximise the ability to detect the multiple risk factors under investigation. Recruitment rates during this biased period were similar (average ten per week) to rates during the initial 12-month recruiting phase (average seven per week). A slightly higher recruitment during the final two months may have been due to a reduced commitment for participants, with their involvement only comprising the antenatal battery of questionnaires and not postnatal questionnaires.

In addition to the 367 participants, twenty-one women declined participation citing a lack of time; five women had already given birth (prematurely); four women were uncontactable by phone, post or email; and fifty-one failed to return antenatal questionnaires. From the 367 women who participated antenatally, a sub-sample of 161 women completed postnatal questionnaires, with a further forty-seven who failed to return postnatal questionnaires; two which were undelivered; and one participant was excluded postnatally due to a stillbirth. For practical reasons, women were selected for postnatal follow-up on the basis of their due date occurring three-months prior to the end of the recruitment period, given that postnatal follow-up was at 10–12 weeks postnatally.

### Procedure and design

Three time points for assessment were used. The first was as part of a larger national study, the second and third timepoints constituted this subsidiary study. Prior to commencement of the study, Ethics Approval was granted from two Victorian hospitals, the Angliss and Northern. Informed consent was gained from participants prior to completing questionnaires.

#### 1. Antenatal Screening through Hospitals (26–32 weeks antenatally)

Midwives trained in the Victorian component of the *beyondblue *National Postnatal Depression Program consecutively recruited women at their routine antenatal visit.

#### 2. Antenatal Risk Factors Battery (28–34 weeks antenatally)

After screening, each woman was contacted by telephone and asked to participate in further research by completing additional questionnaires at home.

#### 3. Postnatal Outcome Measures (10–12 weeks postnatally)

Confirmation of a successful birth was required before sending the postnatal outcome measures at 10 weeks.

### Measures

#### Antenatal screening 26–34 weeks

##### Demographics and psychosocial risk factors

The Demographics and Psychosocial Risk Factors Questionnaire is a four page structured questionnaire gathering demographic and psychosocial information utilised in the larger National Postnatal Depression Program for which results are reported in a separate study [1]. However, for this study we extracted information on age, ethnicity, marital status, income, number of children, history of depression, history of miscarriage or abortion, recent major life events and childhood abuse (emotional, sexual and physical).

##### Edinburgh Postnatal Depression Scale (EPDS)

The Edinburgh Postnatal Depression Scale (EPDS: [36]) was developed as a screening device for perinatal depression. Scores above 9 indicate 'possible depression' while scores above 12 indicate 'probable depression' [37]. For this study, the EPDS was used as an initial screening measure with a cut-off score of 12.5, on the basis of a large Australian population study [38].

#### Antenatal risk factors battery 28–36 weeks

##### Beck Depression Inventory (BDI)

Three versions of the Beck Depression Inventory (BDI: [39]) have been developed, with the original used in this study. Cut-off scores used in research literature have ranged from 8.5 to 16.5 [40-42] with two major Australian based studies employing a 12.5 cut-off score for clinical diagnosis [38,43]. A higher 16.5 cut-off has been traditionally used in estimating the threshold between mild to moderate depression and a more moderate to severe depression. The BDI was used as the depression measure for all analyses.

##### Beck Anxiety Inventory (BAI)

The Beck Anxiety Inventory (BAI: [44]) measures symptom severity of anxiety. While the excellent psychometric properties of the BAI have been well documented, there has been little documented use of the BAI in antenatal populations.

##### Attributional Style Questionnaire (ASQ)

The Attributional Style Questionnaire (ASQ) contains 12 hypothetical situations comprising six positive and six negative events [45]. Only the six negative events were used, as they have stronger psychometric properties and are more strongly linked to depression [45]. Psychometric properties have been previously reported in antenatal samples [40,42].

##### Rosenberg Self-Esteem Scale (RSES)

The Rosenberg Self-Esteem Scale (RSES: [46]) comprises 10 items: five positively worded and five negatively worded. Scores range from 10 to 40, where high scores indicate high self-esteem. The RSES has demonstrated sound overall reliability and validity [47].

##### Social Provisions Scale (SPS)

The Social Provisions Scale (SPS: [48] comprises 24 items and has been used in research investigating associations between social support and postnatal depression [49], and support in the transition to parenthood [50]. Sound psychometric properties have been reported [51,52].

#### Postnatal measures 10–12 weeks

##### Beck Depression Inventory

Described above.

##### Parenting Stress Index (PSI)

The Parenting Stress Index (PSI) [53] measures the level of stress within the parent-child system. It was developed as a screening and diagnostic instrument for those at risk for the development of dysfunctional parenting and behavioural or emotional problems in children. The PSI consists of 101 items and has two domains: child (47 items) and parent (54 items). The child domain items relate to temperament and assess the degree to which each child characteristic causes stress to the parent. The child domains are: (1) adaptability, (2) acceptability, (3) demandingness, (4) mood, (5) distractability/hyperactivity, (6) reinforces parent. The parent domain items assess personal characteristics and level of social support. The parent domains are: (1) depression, (2) attachment, (3) restriction of role, (4) sense of competence, (5) social isolation, (6) relationship with spouse, (7) parent health. Total scores (used in this study) range from 101 to 505. Scores below 175 are considered low, 180–250 is within the normal range and scores above 260 are considered high. The PSI demonstrates good overall psychometric properties [53].

## Results

### Sample

A total of 367 women completed antenatal questionnaires, with a subsample of 161 also completing postnatal questionnaires. The postnatal subsample did not significantly differ from the rest of the antenatal sample on: age (χ^2 ^(27) = 37.96, ns), marital status (χ^2 ^(4) = 2.70, ns) or income level (χ^2 ^(5) = 5.46, ns). There were no significant differences between the two hospital recruitment groups on age (χ^2 ^(27) = 38.10, ns), marital status (χ^2 ^(4) = 8.42, ns), income level (χ^2 ^(5) = 8.33, ns) or the BDI (*t *(365) = -1.26, ns). Thus, the two groups were combined for all analyses.

Descriptive analyses from the antenatal data (N = 367) revealed participants ranged in age from 17 to 45 (M = 30.8, SD = 5.1). The majority of women were either married (n = 243, 66.2%) or in a defacto relationship (n = 109, 29.7%). Six women (1.6%) reported being without a partner. For most, the current pregnancy was either their first (n = 131, 35.7%) or second child (n = 148, 43%). The majority were born in Australia (n = 321, 87.5%). The major ethnic groups were from Europe and America (North and South) and Asia. Annual family income was grouped into six categories: less than $20, 000 (8.7%); $20, 001–$40, 000 (23.7%); $40, 001–$60, 000 (32.7%); $60, 001–$80, 000 (19.9%); greater than $80, 000 (6.8%); and did not wish to divulge (8.2%).

Postnatally depressed and nondepressed groups significantly differed on the Parenting Stress Index (PSI). The PSI contains a 9-item depression subscale. To ensure this subscale did not significantly influence the observed differences between the depressed and nondepressed groups, analyses were conducted with the depression subscale removed. Analyses revealed that even without the depression subscale there was a significant difference between the depressed and non-depressed groups on PSI scores (*t *(159) = -6.13, *p *< .001). The depression subscale was therefore retained for all subsequent analyses, keeping the questionnaire intact to maximise psychometric status.

### Prevalence estimates

The BDI was used to determine the point prevalence of depression antenatally and postnatally. Given the method of recruitment was biased toward including all depressed women in the final months of recruiting, all participants recruited in the biased period of the study, regardless of depression status, were excluded from prevalence analyses. This eliminated 89 women out of 367 leaving 278 women.

#### Prevalence of perinatal depression

Prevalence estimates for those likely to have a moderate to severe clinical depression were classified using a BDI cut-off score of 16.5. At this delineation the point prevalence of depression at 28–32 weeks antenatal was 16.9% (N = 278) and at 10–12 weeks postnatal was 11.2% (N = 161).

#### Prevalence of antenatal anxiety

The overall prevalence of a moderate-severe anxiety in pregnancy, as defined by BAI scores of 16 or greater, was 27.7%. As a further breakdown, 43.5% of women had an absence of anxiety, 28.8% reported mild anxiety symptoms, 21.2% experienced moderate anxiety levels, and 6.5% experienced severe anxiety in pregnancy according to BAI scores.

### Primary data analyses

Regression analyses were conducted to determine the significant predictors for each of the three outcome measures. An examination of the intercorrelations between the variables preceded all regressions. Multicollinearity was examined and found not to be present in any of the analyses.

#### Predictors of antenatal depression

A multiple regression predicting antenatal depression was conducted and statistics are provided in Table 1. Antenatal depression was significantly predicted from the set of dependent variables, (*F*(12, 361) = 101.79, *p *< .001). The significant predictor variables in the regression explained 78% of the variation in antenatal depression. Seven risk factors emerged from the model as significant predictors with the largest being low self-esteem (*β *= -.34, *p *< .001), antenatal anxiety (*β *= .32, *p *< .001) and social support (*β *= -.18, *p *< .001).

**Table 1:** Results of multiple regression for predictors of antenatal depression

Predictor Variables	B	β	sr^2^	t	p
Age	.02	.01	.00	.36	.72
Income	-.36	-.05	-.11	-2.06	.04*
Education	-.26	-.02	-.03	-.85	.39
History of Depression	-.02	-.01	-.00	-.23	.82
History of Abuse	.26	.06	.00	2.22	.03*
History of Miscarriage/Abortion	-.46	-.03	-.05	-1.03	.34
Antenatal Anxiety	2.15	.32	.06	9.46	.00***
Major Life Events	.97	.07	.00	2.55	.01*
Negative Cognitive Style	.04	.11	.00	2.68	.00**
Self-Esteem	-.43	-.34	-.33	-6.46	.00***
Social Support	-1.86	-.18	-.19	-3.71	.00***
R = .88					
R^2 ^= .78***					
Adjusted R^2 ^= .77					
Standard Error = 4.30					

*p < .05, **p < .01, ***p < .001.

B = unstandardised regression coefficients, β = standardised regression coefficients, sr^2 ^= semipartial correlations

#### Predictors of postnatal depression

Given the chronological time factor in which the variables were assessed, a hierarchical regression was performed where antenatal factors were entered followed by the postnatal variable parenting stress. Table 2 displays the results. As antenatal depression has been shown to be a strong predictor of postnatal depression it was entered at stage one. Demographic, historical factors, antenatal anxiety and stressors, and personality factors were entered at stage two and parenting stress at stage three. The first stage explained 51% of the variation in postnatal depression scores, (*F*(1, 156) = 163.37, *p *< .001) and confirmed antenatal depression as a significant predictor of postnatal depression (*β *= .72, *p *< .001). Introducing the additional factors explained an additional 9% of variance and was a significant improvement in model fit, (*F*(12, 144) = 2.52, *p *< .001). Of the variables in the second stage, history of depression (*β *= .19, *p *< .01) and antenatal anxiety (*β *= .18, *p *< .05) were identified as significant predictors of postnatal depression. When parenting stress was added the model was improved significantly, (*F*(1, 143) = 27.84, *p *< .001) and the variance increased by a further 6%. The addition of parenting stress in the third model rendered antenatal anxiety non-significant. When all predictors are included in the model, antenatal depression (*β *= .47, *p *< .001), parenting stress (*β *= .32, *p *< .001) and history of depression (*β *= .15, *p *< .01) are identified as significant predictors of postnatal depression and explain 66% of the variation in postnatal depression scores.

**Table 2:** Results of hierarchical regression for predictors of postnatal depression

Predictor Variables	B	β	sr^2^	t	P
**Model 1**	*F*(1, 156) = 163.37, *p *< .001 *R*^2 ^= .51				
Antenatal Depression	.74	.72	.51	12.78	.00***

**Model 2**	*F*(11, 145) = 2.72, *p *< .001 *R*^2 ^= .60				
Antenatal Depression	.57	.56	.11	6.24	.00***
Age	-.01	-.03	-.06	-.55	.49
Education	.14	.09	.01	1.52	.14
Income	-.05	-.05	-.08	-.79	.45
History of Depression	.10	.19	.03	3.20	.00**
History of Abuse	.06	.09	.01	1.58	.11
History of Miscarriage/Abortion	-.16	-.10	-.18	-1.70	.10
Major Life Events	-.02	-.01	.02	-.21	.83
Antenatal Anxiety	.18	.18	.02	2.47	.02*
Negative Cognitive Style	-.00	-.05	-.08	-.79	.40
Self-Esteem	.00	.02	.00	.19	.85
Social Support	-.08	-.04	-.05	-.50	.57

**Model 3**	*F*(1, 144) = 27.83, *p *< .001				
Antenatal Depression	.48	.47	.07	5.70	.00***
Age	-.01	-.03	-.04	-.62	.54
Education	.12	.08	.01	1.43	.15
Income	-.03	-.03	-.05	-.46	.65
History of Depression	.08	.15	.02	2.69	.00**
History of Abuse	.05	.07	.01	1.46	.15
History of Miscarriage/Abortion	-.15	-.09	-.17	-1.66	.10
Major Life Events	.06	.03	.00	.54	.58
Antenatal Anxiety	.12	.12	.01	1.69	.09
Negative Cognitive Style	-.00	-.03	-.04	-.46	.69
Self-Esteem	.01	.05	.00	.73	.49
Social Support	-.01	-.00	-.00	-.12	.91
Parenting stress	.01	.32	.07	5.28	.00***
R = .81					
*R*^2 ^= .66***					
Adjusted *R*^2 ^= .63					
Standard Error = .75					

*p < .05, **p < .01, ***p < .001.

B = unstandardised regression coefficients, β = standardised regression coefficients, sr^2 ^= semipartial correlations

#### Antenatal depression as a mediator of postnatal depression

Contrary to expectation, many of the variables were found not to be significant predictors of postnatal depression in the final regression analysis. Given that antenatal depression was the strongest predictor of postnatal depression and many of the risk factors were predictive of antenatal depression (Regression 1, Table 1), antenatal depression was examined for its potential mediational effects between the remaining risk factors and postnatal depression.

A variable is considered a mediator when it carries the influence of a given predictor (independent variable: IV) to a given criterion (dependent variable: DV) and accounts for the relationship between the two. Mediation can be tested informally through a series of regressions. If significant, the Sobel test provides a statistical method to assess the significance of the mediator in relation to the IV and DV [54].

Table 3 presents the results for the assessment of antenatal depression as a mediator between risk factors and postnatal depression. Regression coefficients were estimated from each independent variable to postnatal depression. Age, education and history of miscarriage/abortion were not significantly predictive, excluding them from the Sobel test. All other variables satisfied the informal criteria for judging mediation. In each case the path between the risk factors and postnatal depression was reduced to almost zero and statistical non-significance when antenatal depression was included, indicating antenatal depression is a single dominant mediator [54].

**Table 3:** Results of regressions and sobel test for antenatal depression as a mediator for postnatal depression

Predictor Variables	β without Antenatal Depression	β with Antenatal Depression	Sobel Test z-score
Income	-.20**	-.02	-4.13***
History of Abuse	.22**	.11	5.23***
Major Life Events	.24**	.06	5.66***
Antenatal Anxiety	.62***	.08	8.47***
Negative Cognitive Style	.41***	.01	10.35***
Self-Esteem	.49***	.03	-10.28***
Social Support	-.42***	-.01	-10.42***

**p < .01, ***p < .001

In order to calculate the Sobel test for significance, unstandardised regression coefficients and their standard errors were calculated for paths ***a ***and ***b ***for each of the predictor variables. Results revealed that each predictor variable for postnatal depression was significantly mediated by antenatal depression to a *p*-value of < .001, as shown in Table 3.

#### Predictors of parenting stress

A correlation between postnatal depression and parenting stress was established (*r *= .60, *p *< .001). Thus, a hierarchical regression was conducted entering postnatal depression in the final stage in an attempt to unveil other variables that may contribute to the prediction of parenting stress that may be hidden by the overpowering postnatal depression variable. Table 4 displays the results. The range of predisposing factors and antenatal stressors were entered at stage one, antenatal depression was entered at stage two followed by postnatal depression at stage three. The first stage explained 33% of the variation in parenting stress scores, (*F*(11, 145) = 6.52, *p *< .001) and identified antenatal anxiety (*β *= .32, *p *< .001) and low self-esteem (*β *= -.24, *p *< .05) as significant. Introducing antenatal depression explained an additional 3% variance and was a significant improvement in model fit, (*F*(12, 144) = 6.74, *p *< .001), however low self-esteem was rendered as non-significant. When postnatal depression was added the variance increased by another 11% and again improved the model fit significantly, (*F*(13, 143) = 9.79, *p *< .001). The addition of postnatal depression rendered antenatal depression and anxiety as non-significant. Thus, the strongest predictor of parenting stress was postnatal depression.

**Table 4:** Results of hierarchical regression for predictors of parenting stress

Predictor Variables	B	β	sr^2^	t	p
**Model 1**	F(10, 147) = 6.82, p < .001 R^2 ^= .32				
Age	.06	.01	.00	.09	.94
Income	-3.12	-.10	-.02	-1.40	.31
History of Depression	1.49	.10	.01	1.30	.15
History of Abuse	1.03	.06	.00	.76	.39
History of Miscarriage/Abortion	1.82	-.04	-.07	-.50	.74
Major Life Events	-6.12	-.11	-.20	-1.47	.13
Antenatal Anxiety	9.15	.32	.07	3.91	.00***
Negative Cognitive Style	-.10	-.05	-.08	-.56	.64
Self-Esteem	-1.80	-.24	-.33	-2.43	.04
Social Support	-5.23	-.09	-.12	-.90	.20

**Model 2**	*F*(1, 146) = 6.56, *p *< .001 *R*^2 ^= .35				
Age	-.16	-.02	-.04	-.28	.81
Income	-2.57	-.09	.16	-1.17	.43
History of Depression	1.73	.11	.01	1.53	.10
History of Abuse	.87	.05	.00	.65	.47
History of Miscarriage/Abortion	-1.78	-.04	-.07	-.50	.72
Major Life Events	-6.99	-.12	-.23	-1.70	.08
Antenatal Anxiety	5.73	.20	.02	2.15	.03*
Negative Cognitive Style	-.22	-.10	-.15	-1.15	.25
Self-Esteem	-1.07	-.14	-.18	-1.37	.28
Social Support	-4.52	-.07	-.11	-.80	.25
Antenatal Depression	8.75	.29	.03	2.55	.01*

**Model 3**	*F*(1, 145) = 28.18, *p *< .001				
Age	-.07	-.01	-.02	-.04	.97
Income	-2.10	-.07	-.13	-.61	.54
History of Depression	.31	.02	.00	.50	.62
History of Abuse	-.07	-.00	-.01	-.05	.96
History of Miscarriage/Abortion	.72	.01	.00	.36	.72
Major Life Events	-6.94	-.12	-.22	-1.90	.06
Antenatal Anxiety	2.95	.10	.01	1.23	.22
Negative Cognitive Style	-.15	-.07	-.11	-.77	.45
Self-Esteem	-1.22	-.16	-.21	-1.36	.17
Social Support	-3.20	-.05	-.07	-1.01	.32
Antenatal Depression	-.24	-.01	-.01	-.02	.98
Postnatal Depression	15.51	.52	.11	5.31	.00***
R = .67					
*R*^2 ^= .45***					
Adjusted *R*^2 ^= .41					
Standard Error = 28.34					

*p < .05, ***p < .001.

B = unstandardised regression coefficients, β = standardised regression coefficients, sr^2 ^= semipartial correlations

#### Postnatal depression as a mediator of parenting stress

As with the results for postnatal depression, many of the predictor variables for parenting stress were found not to be significant. Given that postnatal depression was the only significant predictor of parenting stress it was examined for its potential mediational effects between the remaining risk factors.

Table 5 presents the results. Regression coefficients were estimated from each independent variable to parenting stress. Age, education, income, history of abuse, history of miscarriage/abortion and major life events were not significantly predictive of parenting stress and so were not included in further mediational analyses. All other variables satisfied the informal criteria for judging mediation. In each case the path between the risk factors and parenting stress was reduced to almost zero and statistical non-significance when postnatal depression was included.

**Table 5:** Results of regressions and sobel test for postnatal depression as a mediator for parenting stress

Predictor Variables	β without Postnatal Depression	β with Postnatal Depression	Sobel Test z-score
History of Depression	.26**	.05	3.99***
Antenatal Anxiety	.46***	.10	5.31***
Negative Cognitive Style	.29***	.03	4.68***
Self-Esteem	-.45***	-.07	-5.03***
Social Support	-.40***	.14	-4.97***

**p < .01, ***p < .001

The Sobel test for significance revealed that each predictor variable for parenting stress was significantly mediated by postnatal depression to a *p*-value of < .001, as shown in Table 5.

## Discussion

Most of the previously established risk factors [1] played a role in predicting antenatal depression, postnatal depression and parenting stress.

An impressive 78% of the variance of antenatal depression was explained by seven factors: low self-esteem, antenatal anxiety, low social support, negative cognitive style, major life events, low income and a history of abuse. Age, education and depression history were not significant in the regression but were significantly correlated with antenatal depression.

Antenatal depression, history of depression and concurrent parenting stress accounted for 66% of the variance in explaining postnatal depression. Furthermore, antenatal depression was revealed as a dominant mediator between seven risk factors and postnatal depression, namely antenatal anxiety, major life events, low self-esteem, low social support, negative cognitive style, history of abuse and low income. Age was not found to be significant in the regression but was correlated with postnatal depression. Education was not significantly related to postnatal depression.

The only identified factor for parenting stress was concurrent postnatal depression, which alone accounted for 45% of the variance. None of the antenatal risk factors were directly predictive of parenting stress. However, postnatal depression was revealed as a dominant mediator between five risk factors and parenting stress: antenatal anxiety, low self-esteem, low social support, negative cognitive style and history of depression. While antenatal depression did not contribute to the prediction of parenting stress, even through the mediation of postnatal depression, antenatal depression was found to be significantly related to parenting stress. Age, education, income, history of abuse and major life events were not significantly related to parenting stress.

Limitations of this study include the under-representation of women who are not partnered or from diverse cultures in the sample and subsample, which largely comprised married, Australian-born women. Compared with Victorian averages from 2004 [55] this sample comprised a higher percentage of partnered (95.9% this sample; 86.5% Victorian average) and Australian-born women (87.5% this sample; 76.1% Victorian average). Thus, the results of this study may have limited generalisability to women from other cultures or unpartnered women. Those categorised in the antenatal depressed group were assumed to have their depressive onset in pregnancy but this was not established. As such, some women identified as depressed antenatally may have been depressed prior to conception. Previous research found that time of depressive onset, prior to or during pregnancy, was related to the duration of the depressive episode [7]. Multiple risk factors for antenatal depression were measured concurrently in the antenatal period raising two conceptual limitations. First, pervasive negative reporting may have occurred with numerous concurrent measures being completed by those currently depressed. Second, the interpretation of significant risk factors as truly predictive of antenatal depression is limited given the risk factors were measured concurrently rather than prospectively. Similarly, postnatal depression and parenting stress were measured concurrently raising limitations about a genuine predictive relationship. The overlap between the constructs of postnatal depression and the PSI, as previously acknowledged, further limits the ability to interpret results. However, an attempt was made to minimise the confounding of results by confirming that observed differences on the PSI between depressed and non-depressed group were not solely due to the depression subscale. Finally, although this study assessed many risk factors, it is a challenge to account for all previously identified variables in any one multivariate study. Caregiving history including a harsh, rejecting parenting style in one's family of origin and attachment styles have been linked with antenatal and/or postnatal depression [56,57] and were not accounted for in this study.

These limitations provide future research directions. Most notably, onset of depression, prior to or during pregnancy, may relate to duration of the depressive episode. Additionally, research into effective interventions for antenatal depression in an effort to diminish or ameliorate postnatal depression and early parenting stress seem warranted.

### Integrative model of risk factors for antenatal depression, postnatal depression and parenting stress

**Figure 1:**
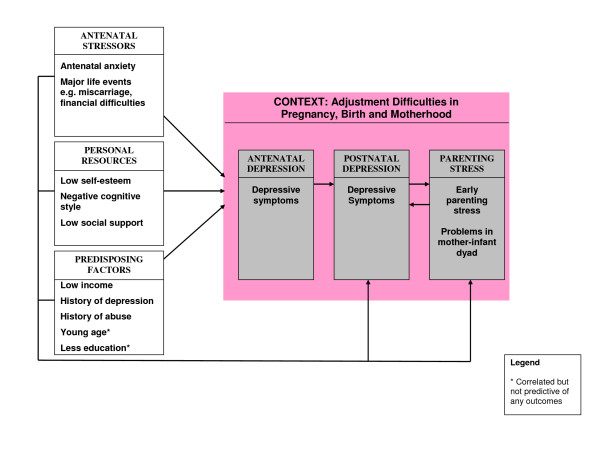
A psychosocial model of antenatal depression, postnatal depression and parenting stress.

In previous work, we conceptualised a biopsychosocial model of postnatal depression, which comprised vulnerability factors, precipitating factors, maintaining factors and considered there may be some mediating factors in the presentation of postnatal depression [29]. Here, we propose a broader contextual model of adjustment in pregnancy, birth and motherhood. We highlight the importance of antenatal stressors, personal resources and predisposing factors in the development and maintenance of antenatal depression and subsequent postnatal depression and parenting stress (Figure 1). The three outcome measures are embedded within the context. This model is an overall schematic representation of the results from this study and was not specifically tested. It does not take into account the relative weighting of each risk factor variable in relation to the three outcome measures.

The strongest predictor of postnatal depression was antenatal depression, which also served as a mediator between many risk factors. Similarly, postnatal depression was the strongest predictor of parenting stress and postnatal depression also served as a mediator between many risk factors. These relationships are depicted by the suggested linear progression from the multiple risk factors to antenatal depression, which is then predictive of postnatal depression, which in turn predicts parenting stress. The relationship between the three outcome measures is important in creating an integrative risk profile, as seen in Figure 1.

## Conclusion

The importance of antenatal depression has been largely under recognised with the focus of research and treatment programs on postnatal depression. Yet results from this study are consistent with earlier findings indicating that antenatal depression is the strongest risk factor for postnatal depression [1,12-14,24,58]. Antenatal depression was also found here to be a mediator between many risk factors and postnatal depression.

The relationship between parenting stress and postnatal depression appears to be a reciprocal one, with each contributing to the other. While significant antenatal risk factors were predictive of postnatal depression, parenting stress appeared to be predicted exclusively by postnatal depression with postnatal depression also operating as a mediator between the antenatal risk factors and parenting stress.

Targeted interventions for antenatal and postnatal depression may reduce both the symptom severity and incidence of perinatal depression and assist in the amelioration or prevention of early parenting stress. Given the enormous public health impact of perinatal depression, implementation and empirical validation of targeted antenatal interventions are proposed for future research.

## Competing interests

The author(s) declare that they have no competing interests.

## Authors' contributions

BL and JM equally contributed to the conceptualisation and design of the study. BL carried out data collection and analyses under the supervision of JM. The manuscript was initially drafted by BL. Both BL and JM reviewed and contributed to the submitted manuscript. Both authors read and approved the final manuscript.

## Pre-publication history

The pre-publication history for this paper can be accessed here:


